# Functional Outcomes of Intensive Rehabilitation Versus Fusion Surgery in Patients With Low Back Pain Due to Lumbar Degenerative Spine Disease: A Systematic Review and Meta-Analysis

**DOI:** 10.7759/cureus.102509

**Published:** 2026-01-28

**Authors:** Tito Guillermo D Rejante, Kevin Paul B Ferraris, Joseph Erroll V Navarro, Kenny S Seng, Jose Carlos S Alcazaren

**Affiliations:** 1 Section of Neurosurgery, Jose R. Reyes Memorial Medical Center, Manila, PHL

**Keywords:** fusion surgery, low back pain, lumbar spine, rehabilitation, spondylolisthesis

## Abstract

Lumbar degenerative disease is mostly managed non-operatively, but lumbar spinal fusion has been used for almost a century. It has shown clinical efficacy in decreasing pain and disability scores and allowing patients to return to work. However, spinal fusion may not be clinically effective and may place a financial burden on patients. The alternative to spinal fusion is physical therapy and rehabilitation for lumbar degenerative diseases.

This study aims to compare the functional outcomes of intensive rehabilitation and lumbar spine fusion surgery among patients with chronic low back pain from lumbar spine degenerative disease. We conducted a systematic search of clinical trials on the topic, followed by a meta-analysis using a random-effects model.

The functional outcomes compared included the Oswestry Disability Index (ODI) score, improvement in low back pain and leg pain, as well as overall patient outcome. Five randomized controlled trials were included in the meta-analysis. There was a 7.25-point improvement in the change in the ODI (95% CI: 1.22-13.17; p = 0.02; I² = 98%), favoring fusion surgery. All studies showed improvement in low back pain, with a VAS score change of 11.49, favoring fusion surgery (95% CI: 4.48-18.50; p = 0.001; I² = 96%). There was a Visual Analog Scale (VAS) score change of 7.2 in leg pain improvement (95% CI: -8.58 to 22.97; p = 0.37; I² = 98%); however, the effect was not significant. There was no significant difference in terms of overall patient outcome (95% CI: 0.23-1.08; p = 0.08; I² = 78%).

Among patients with chronic low back pain, lumbar spine fusion surgery showed improvement in functional outcomes related to changes in disability and low back pain when compared with intensive rehabilitation. However, the two treatment options showed no differences with respect to improvement in leg pain and overall patient outcome.

## Introduction and background

Back pain is widespread, with over 600 million individuals afflicted, and is the leading cause of years of disability worldwide [1]. Low back pain is the hallmark of lumbar degenerative disease; unfortunately, it is a nonspecific complaint when trying to determine which patients will benefit most from surgery [2]. Initial management is mostly non-operative, but lumbar spinal fusion has been used for almost a century. It has shown clinical efficacy in decreasing pain and disability scores and allowing patients to return to work [2]. The principle of spinal fusion is to provide a biomechanically lasting interbody union, which can be accomplished using different surgical approaches, implants, and grafts [3]. However, fusion procedures have not been effective for all patients, and the alternative is physical therapy and rehabilitation.

Physical rehabilitation is the most common method used to apply non-operative treatment of symptoms in patients with chronic low back pain. Therapeutic protocols may include modalities for pain relief, bracing, exercise, ultrasound, electrical stimulation, and activity modification. Physical rehabilitation is recommended to reduce pain, restore range of motion and function, strengthen and stabilize the spine, and restore neural tissue mobility [4,5]. In low- and middle-income countries, where the costs of spinal implants could be prohibitive for the majority of patients with chronic low back pain, this study becomes relevant in determining whether intensive rehabilitation may become a reasonable option.

In randomized controlled trials (RCTs) by Brox et al. [6], Fairbank et al. [7], and Mannion et al. [4], there were no statistically significant differences between treatment groups randomized to either lumbar fusion surgery or cognitive intervention and exercises. In contrast, the RCTs by Möller and Hedlund [8] and Fritzell et al. [9] showed that patients randomized to the surgical group had better outcomes than their non-surgical counterparts. For clinicians who deliberate and contemplate these two diverging treatment options, it becomes imperative to clarify the weight of the evidence through a meta-analysis.

This article was previously shared as a preprint on the Research Square server on April 29, 2021 (https://doi.org/10.21203/rs.3.rs-478001/v1).

## Review

Materials and methods

Search Strategy

We conducted a systematic review and meta-analysis of prospective studies and RCTs published between January 2000 and June 2020. A detailed search was conducted in PubMed, the Cochrane Central Registry of Clinical Trials, and EMBASE using the following keywords: “chronic low back pain,” “lumbar spine,” “degenerative disease,” “spinal fusion,” “lumbar fusion,” “surgical stabilization,” “physical therapy,” and “rehabilitation.” An effort was made to search for relevant grey literature through OpenGrey, BASE, and CORE.

Eligibility Criteria

The study types included in our research were both RCTs and prospective studies. RCTs comparing the outcomes between groups of patients with chronic low back pain for more than one year from degenerative disc disease or spondylosis (with or without spondylolisthesis) treated with either fusion surgery or rehabilitation, and studies with random allocation between groups, were considered eligible for this study. Functional outcomes included the Oswestry Disability Index (ODI), low back pain, leg pain, and overall patient outcome. There should be at least a one-year follow-up.

Exclusion criteria included case reports, letters to the editor, commentaries, cross-sectional surveys, and documentaries. Trials involving patients with low back pain due to fracture, metastasis, or inflammation, as well as studies comparing different forms of surgical interventions and studies with different outcomes of interest, were excluded. Data collection was performed by two independent authors in accordance with the mentioned eligibility criteria. Any discrepancies were resolved through collaborative discussion.

Study Selection and Reporting

All included articles were independently screened and assessed for validity and eligibility. The appraisal was done using the Cochrane Methodological Risk Assessment Tool. The following data were extracted from each of the included trials: author, year of publication, type of population, study design, sample size, duration of study, intervention, comparator, study outcomes, and location of population. Our report followed the generally accepted guideline, the Preferred Reporting Items for Systematic Reviews and Meta-Analyses (PRISMA).

Statistical Analysis

Data were extracted from tables and the associated text describing the outcomes before and after treatment. The functional outcomes were pretreatment and posttreatment differences based on the ODI, low back pain, or leg pain, determined through the Visual Analog Scale (VAS) score, and overall patient outcome. The homogeneity of odds ratios (ORs) was tested using Cochran's Q statistic. If homogeneity was rejected at the 0.1 level, the ORs were analyzed using the random-effects model, with the presumption that multiple potential sources of heterogeneity were present in the included studies. The overall ORs were computed using the Cochran-Mantel-Haenszel method. Meta-analysis results were presented as mean differences (MDs) and standard deviations (SDs), with 95% confidence intervals (CIs), and were graphically displayed as forest plots. The inverse variance method was used to calculate estimates for continuous variables. Review Manager version 5.4 (The Cochrane Collaboration, Copenhagen, Denmark) was the software used for the analyses.

Results

Search Results

**Figure 1:**
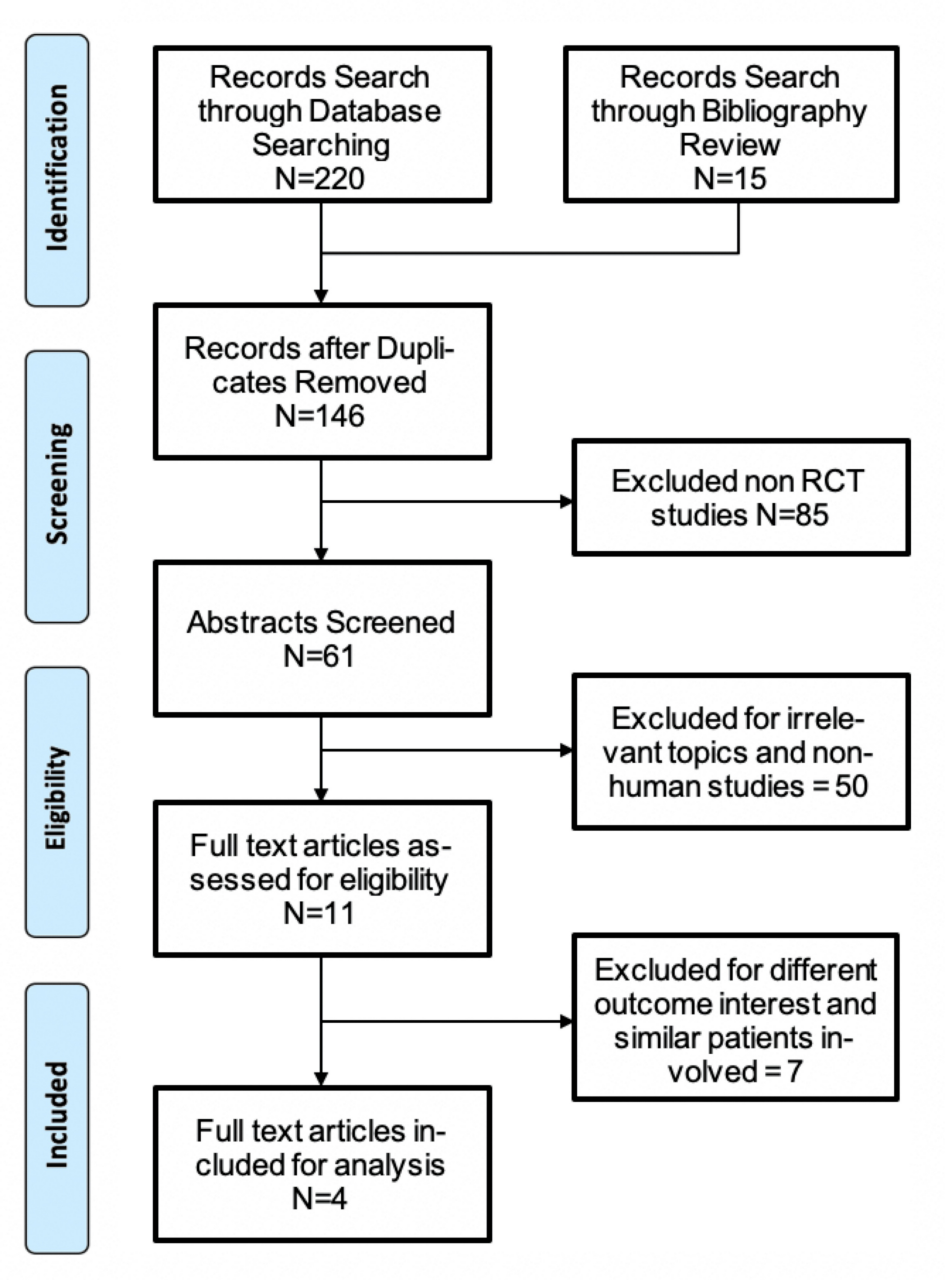
Flow diagram for data extraction strategy in conformity with the PRISMA guidelines PRISMA, Preferred Reporting Items for Systematic Reviews and Meta-Analyses

The search revealed 220 articles from a database search and 15 articles from a bibliography search. After 89 duplicates and 85 non-RCTs were excluded, 61 articles were screened, and full-text articles were reviewed. Of the 11 screened-in articles, seven were further excluded because they had different outcome interests and similarities in the patients involved. Four studies fulfilled the eligibility criteria and were analyzed after a full-text systematic review. Figure 1 shows the flow of the selection process, while Table 1 summarizes the characteristics of the included studies.

**Table 1:** Summary of the characteristics of the included studies

Study	Randomised patients (surgery/rehabilitation or physical therapy)	Intervention	Follow-up interval	Inclusion criteria	Setting	Tool	Endpoints	Reference
Fairbank et al. (2005)	176/173	Intensive rehabilitation/spinal fusion surgery	2 years	Aged between 18 and 55, with more than a 12-month history of chronic low back pain (with or without referred pain), and irrespective of whether they had had previous root decompression or discectomy	United Kingdom	Oswestry disability index and the shuttle walking test, SF-36 questionnaire	No difference	[7]
Fritzell et al. (2001)	72/222	Three types of spinal fusion surgery (posterolateral fusion with variable screw placement and interbody bone graft)/physical therapy (nonsurgical)	6 months, 12 months, 2 years	Aged 25-65 years and of both sexes with severe chronic low back pain may have had previous spine surgery, except for successful removal of a herniated disc more than 2 years before entering the study, and with no persistent nerve root symptoms	Sweden	Oswestry disability index, visual analog scale (VAS), general function score (GFS), and Zung depression scale	Decrease in disability, pain, and depression, and the overall result was significantly greater in the surgical group	[9]
Mannion et al. (2013)	242/231	Spinal fusion surgery/multidisciplinary cognitive-behavioral and exercise rehabilitation	2 years, 11 years	Aged 18-55 years (UK), 25-60 years (Norway); low back pain duration of at least 1 year	United Kingdom and Norway	Oswestry disability index score	No statistically or clinically significant differences between treatment groups for ODI scores	[4]
Brox et al. (2003)	35/26	Lumbar fusion surgery/cognitive Intervention and exercises	1 year	Age 25-60 years. Low back pain duration for at least 1 year	Norway	Oswestry disability index score	Equal improvement in patients with chronic low back pain and disc degeneration randomized to cognitive intervention and exercises, or lumbar fusion	[6]

Study Characteristics

The four studies included were published from 2000 to 2020, involving 1,177 patients. Studies took place in the United Kingdom, Sweden, and Norway. All of the studies involved patients with chronic low back pain. All studies had lumbar fusion as the main surgical approach, described as posterolateral fusion, compared with rehabilitation and physical therapy. The non-surgical approach, which includes rehabilitation, physical therapy, and exercises, had different programs and routines across the studies. Follow-up ranged from 1 year to 11 years. Four of the studies had a follow-up period of two years. All of the studies used the ODI to measure changes in disability. Four studies used the VAS to measure changes in pain. Most of the studies were single-blinded. There were no significant differences in baseline characteristics of the groups being compared, and all studies reported randomization. Four studies had adequate follow-up. The study by Mannion et al. [4] shows a 45% dropout in their long-term follow-up of 11 years, which may lead to attrition bias. Table 2 gives a summary of the risk-of-bias assessment for the included studies.

**Table 2:** Summary of the risk of bias assessment for the included studies using the Cochrane Methodological Quality Assessment

Criteria	Fairbank et al. (2005) [7]	Fritzell et al. (2001) [9]	Mannion et al. (2013) [4]	Brox et al. (2003) [6]
Randomization sequence generation	Low	Low	Low	Low
Allocation concealment	Low	Low	High	Low
Blinding	High	High	High	High
Incomplete data	Low	Low	High	Low
Selective reporting	Low	Low	Low	Low
Overall RoB	Low	Low	Low	Low
Remarks	High risk for performance bias	High risk for performance bias	High risk for selection, performance, and attrition bias; >Risk for reporting bias	High risk for performance bias

Functional outcomes

Oswestry Disability Index (ODI)

**Figure 2:**
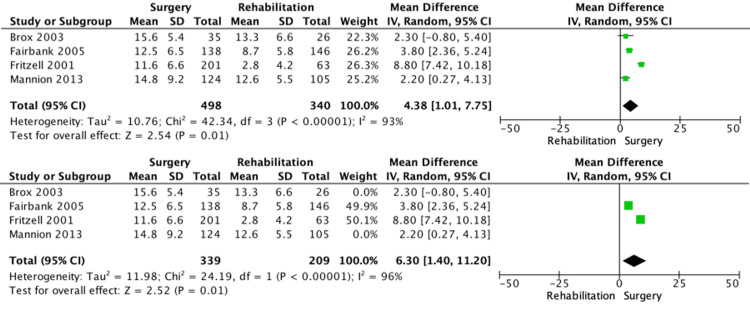
Forest plot Constructed forest plot among patients with chronic low back pain using a random-effects model. Comparator 01: lumbar spine fusion surgery versus rehabilitation. Outcome 01: change in Oswestry Disability Index. Sensitivity analysis among patients with chronic low back pain using a random-effects model. Comparator 01: lumbar spine fusion surgery versus rehabilitation. Outcome 01: change in Oswestry Disability Index at two years. Source: [4,6,7,9]

The forest plot shows the MD in change in ODI between the lumbar spine fusion surgery and rehabilitation groups (Figure 2). All studies showed improvement in the change in ODI, favoring surgery. There was a 4.38 improvement in the change in ODI, with high heterogeneity among the studies (95% CI: 1.01-7.75; p = 0.01; I² = 93%). The sensitivity analysis (Figure 2) shows the MD in change in ODI between the lumbar spine fusion surgery and rehabilitation groups at two years. Studies by Mannion et al. and Brox et al. have 11-year and 1-year follow-ups, respectively [4,6]. All studies showed improvement in the change in ODI after two years, favoring surgery. There was a 6.3 improvement in the change in ODI (95% CI: 1.4-11.2; p = 0.01; I² = 96%).

Low Back Pain

**Figure 3:**
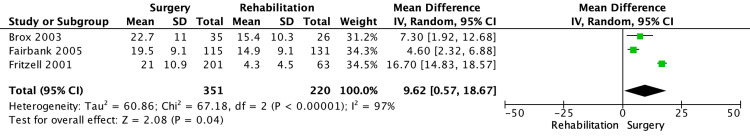
Subgroup analysis of low back pain Subgroup analysis of improvement in low back pain using a random-effects model between lumbar spine fusion surgery and rehabilitation. Comparator 01: lumbar spine fusion surgery versus rehabilitation. Outcome 02: improvement in low back pain. Source: [6,7,9]

The subgroup analysis shows the MD in improvement in low back pain between the lumbar spine fusion surgery and rehabilitation groups (Figure 3). All studies showed improvement in low back pain, favoring surgery. There was a 9.62 improvement in VAS score for low back pain (95% CI: 0.57-18.67; p = 0.04; I² = 97%).

Leg Pain

**Figure 4:**
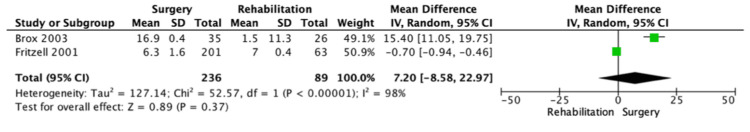
Subgroup analysis of leg pain Subgroup analysis of improvement in leg pain using a random-effects model between lumbar spine fusion surgery and rehabilitation. Comparator 01: lumbar spine fusion surgery versus rehabilitation. Outcome 03: improvement in leg pain. Source: [6,9]

The subgroup analysis shows the MD in improvement in leg pain between the lumbar spine fusion surgery and rehabilitation groups (Figure 4). There was a 7.2 improvement in the VAS score for leg pain (95% CI: -8.58 to 22.97; p = 0.37; I² = 98%); however, the effect was not significant.

Overall Patient Outcome

**Figure 5:**
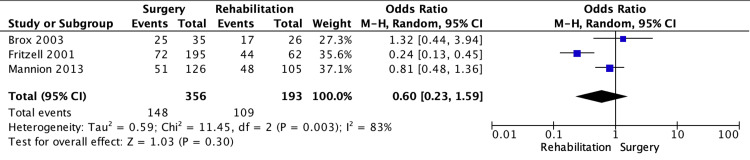
Subgroup analysis of overall patient outcome Subgroup analysis of overall patient outcome using a random-effects model between lumbar spine fusion surgery and rehabilitation. Comparator 01: lumbar spine fusion surgery versus rehabilitation. Outcome 04: overall patient outcome. Source: [4,6,9]

The subgroup analysis shows the OR in overall patient outcomes between the lumbar spine fusion surgery and rehabilitation groups (Figure 5). There was no significant difference in the overall patient outcome (95% CI: 0.23-1.59; p = 0.30; I² = 83%).

Discussion

This meta-analysis of five RCTs showed a significant improvement in disability index scores and low back pain among patients who underwent lumbar spine fusion surgery, compared to those who received intensive rehabilitation. However, these findings were in contrast with the studies of Mannion et al., Brox et al., Fairbank et al., and Fritzell et al. [4,6,7,9], which revealed that intensive rehabilitation is comparable to lumbar fusion surgery. Notably, the two options showed no significant differences in leg pain scores or improvement in overall patient outcome, as confirmed by the patients’ subjective validation of improvement in outcome and reduced pain scores. 

Pain is naturally subjective, and validated pain tools were used across the included studies to quantify pain intensities. Lumbar fusion surgery showed significant improvement in low back pain; however, scores in leg pain did not significantly improve. Additionally, no statistical difference was seen between the two treatments regarding overall patient outcomes. These results align with those of Chou et al. [10], which similarly found intensive rehabilitation to be comparable to fusion surgery in patients with low back pain and lumbar spondylolisthesis. However, patients with lumbar degenerative disease may benefit more from fusion surgery, particularly those presenting with stenosis and evidence of instability. Also, the landmark Spine Patient Outcomes Research Trial (SPORT), with randomized (n = 304) and observational (n = 303) patients with lumbar spondylolisthesis, supports these findings, showing that surgery yielded superior outcomes at two-, four-, and eight-year follow-up [11-13].

The forest plot on the change in the disability score highlighted a rather high heterogeneity of I² = 98% (Figure 2). Many factors may contribute to this variability, such as the duration of the evaluation. To address this variable, a sensitivity analysis included only studies with longer follow-ups of more than two years. Despite this, the heterogeneity remained high, which may suggest that other variables, such as rehabilitation strategies and surgical techniques, influenced the results. In addition, studies of these kinds of treatments, where blinding is difficult, are subject to performance bias, which may have been a contributing factor.

Given the overall findings, lumbar spine fusion surgery appears to offer better outcomes in lowering disability and improving low back pain among patients with chronic low back pain due to degenerative causes, such as spondylolisthesis. These findings are consistent with the results of the study by Möller and Hedlund [8], which demonstrated that surgical management has an advantage over an intensive exercise program in addressing such cases, in terms of improved function and reduced low back pain. Intensive rehabilitation serves as a viable alternative for patients who do not prefer surgery, as it appears comparable to lumbar spinal fusion surgery in improving leg pain and overall patient outcomes. Considering factors such as costs and accessibility, and the presence of comorbid conditions that may contraindicate surgery or general anesthesia, intensive rehabilitation represents a reasonable option for many patients.

## Conclusions

Among patients with chronic low back pain, lumbar spine fusion surgery showed improvement in functional outcomes related to changes in disability and low back pain, when compared with intensive rehabilitation. However, the two treatment options showed no differences with respect to improvement in leg pain and overall patient outcome.
